# Anterior Urethral Metastasis of Prostate Cancer Presenting With Minimal Prostate-Specific Antigen (PSA) Elevation

**DOI:** 10.7759/cureus.96979

**Published:** 2025-11-16

**Authors:** Faria Rahman Antara, Riya Kataria

**Affiliations:** 1 General Surgery, Ashford and St. Peter's Hospitals NHS Foundation Trust, Surrey, GBR; 2 Internal Medicine, Ashford and St. Peter's Hospitals NHS Foundation Trust, Surrey, GBR

**Keywords:** anterior urethral lesion, haematuria, prostate cancer (pca), rare metastasis, urethral metastasis

## Abstract

Prostate cancer metastasising to the urethra is a very rare occurrence of advanced disease. Herein, we present a case of a 64-year-old male patient presenting with urethral metastasis of prostate adenocarcinoma. This patient initially presented in May 2024 with visible haematuria, with flexible cystoscopy in July 2024 revealing a polypoid lesion in the anterior urethra measuring 1.5 cm and a second villous growth in the prostatic fossa. The patient’s initial prostate adenocarcinoma was diagnosed in 2017, managed with transurethral resection of the prostate (TURP), radical radiotherapy, and adjuvant hormonal therapy up until 2019, with stable disease and prostate-specific antigen (PSA) until 2024. Initial management was resection of both lesions with subsequent repeat cystoscopy for further clearance of the anterior urethral lesion. Histopathological analysis of both lesions revealed the lesion in the prostatic fossa to be a nephrogenic metaplasia and the anterior urethral lesion to be prostatic adenocarcinoma (Gleason 4+4). After a multidisciplinary team discussion, the patient was deemed for further hormonal treatment to manage this new metastatic deposit. Continued oncological review and management are still underway. Occult metastasis of prostate cancer in this manner is extremely rare in the literature and requires early detection and clinical vigilance for timely treatment.

## Introduction

Tumours involving the urethra, either primary or metastatic, are extremely rare [1]. Urethral metastasis of prostate cancer typically presents with gross haematuria and lower urinary tract symptoms, the former being most frequently reported, with recurrence generally occurring between one and 13 years post-treatment [2]. Following surgical treatment of prostate cancer, prostate-specific antigen (PSA) can be used as an indicator for identifying residual disease or recurrence [2]; however, due to the infrequent occurrence of the urethral metastasis, the diagnosis can be easily missed or overlooked in patients with stable or low PSA, unless clinicians maintain a high index of suspicion even years after definitive prostate cancer therapy. Herein, we present the case of a man in his 60s who was diagnosed with anterior urethral metastasis originating from prostate cancer, with a history of high-risk localised disease treated seven years earlier. 

This abstract was presented at the Kent, Surrey and Sussex (KSS)/South Thames Regional Urology Meeting in the United Kingdom (oral presentation) on 21 November 2024.

## Case presentation

A British male in his 60s with multiple vascular comorbidities, including type 2 diabetes, hypertension, and prior transient ischaemic attacks, underwent transurethral resection of the prostate (TURP) for high-pressure chronic urinary retention. Histopathology of the resected tissue unexpectedly indicated prostate adenocarcinoma, Gleason score 4+4, staged T1aN0M0, with a post-TURP PSA of 64 ng/ml. Initial staging CT and bone scans showed no evidence of metastasis. He then received radical external beam radiotherapy to the prostate and pelvic nodes along with two years of androgen deprivation therapy. PSA nadired and remained stable for four years. 

Recently, he presented with new-onset visible haematuria. He did not have any associated lower urinary tract obstructive symptoms. Flexible cystoscopy revealed a 1.5 cm polypoid lesion arising from the roof of the anterior urethra, approximately 2-3 cm proximal to the external meatus, and a smaller villous lesion in the prostatic urethra close to the bladder neck. 

Investigations 

**Figure 1:**
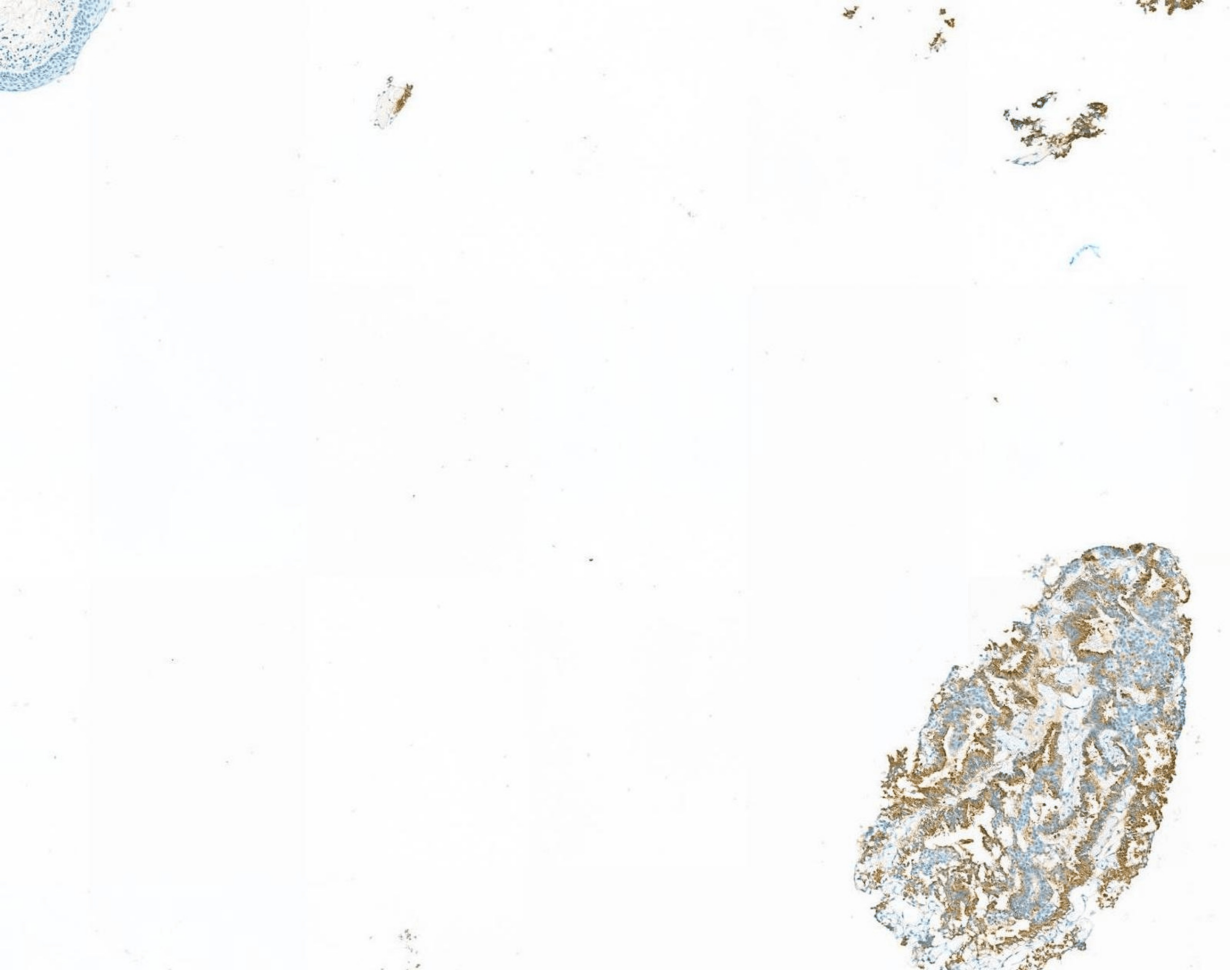
Histology of anterior urethral sample PSA: prostate-specific antigen PSA immunostain (×5) showing cytoplasmic positivity in prostatic-type glands

**Figure 2:**
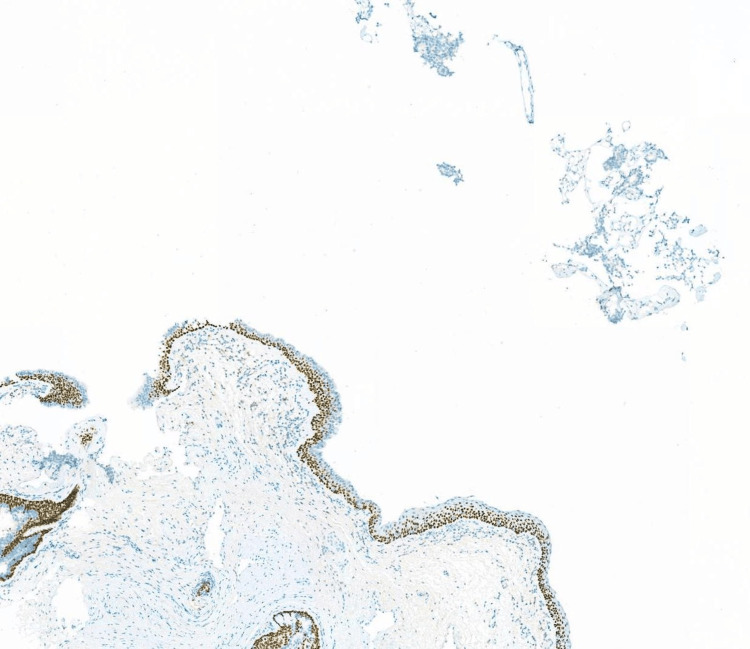
Histology of anterior urethral sample p63 immunostain (×5) showing nuclear positivity in basal cells of urothelium

Rigid cystoscopy with biopsy further confirmed the anterior urethral lesion to be adenocarcinoma of prostatic origin. Immunohistochemistry was positive for PSA and NKX3.1; however, negative for PAX8 and p63. Histological examination of the villous lesion found in the prostatic urethra was suggestive of nephrogenic metaplasia; however, a definitive diagnosis could not be established due to limited tissue in the specimen and the absence of any residual lesion on subsequent endoscopic resection. Histology of the anterior urethral lesion demonstrated a small acinar adenocarcinoma with a Gleason score of 4+4 ( Figures 1, 2).

The latest staging CT showed no nodal or distant disease. Prostate-specific membrane antigen (PSMA) positron emission tomography (PET)/CT also showed focal uptake confined to the prostate gland, without any evidence of systemic spread. 

Treatment 

He underwent an endoscopic resection of the anterior urethral lesion. The tumour, measuring approximately 1 cm and located 2-3 cm proximal to the external meatus, was excised using a monopolar diathermy. It was again confirmed to be metastatic prostatic adenocarcinoma with the help of histology, with immunohistochemistry positive for PSA and prostatic-specific acid phosphatase (PSAP). There were no additional suspicious lesions that were identified. 

Outcome and follow-up 

This incidence was considered an isolated anterior urethral recurrence of prostate adenocarcinoma at the local urology multidisciplinary meeting. Although endoscopic clearance was achieved, it was concluded that complete surgical excision was not technically feasible. In the absence of a systemic spread, recommencement of the androgen deprivation therapy was recommended. The patient was initially reluctant to undergo hormonal treatments but remains under close follow-up with regular PSA monitoring and interval cystoscopy to guide further management. The most recent flexible cystoscopy revealed no evidence of recurrence, and PSA levels continued to remain stable during ongoing follow-up. Table 1 demonstrates a clear timeline of the events over a few years.

**Table 1:** Clinical course, investigations, and interventions TURP: transurethral resection of prostate; EBRT: external beam radiation therapy; ADT: androgen deprivation therapy; PSA: prostate-specific antigen; IHC: immunohistochemistry; NKX3: It is a 234 amino acid transcription factor protein that is expressed in prostate; PAX8: paired box gene 8; p63: tumour protein P63 is a transcription factor of the p53 gene family; PSMA PET/CT: prostate-specific membrane antigen positron emission tomography/computed tomography; PSAP: prostate-specific acid phosphatase; MDT: multidisciplinary team

Year/date	Event/finding	Key results/notes
2017	TURP for high-pressure chronic urinary retention	Histology: adenocarcinoma Gleason 4+4, T1aN0M0, post TURP PSA 64 ng/mL
2017	Staging CT and bone scan	No metastatic disease
2017–2019	Radical EBRT (78 Gy/37 fx) + ADT (2 years)	Completed; PSA stable thereafter
2022	PSA monitoring	0.188 ng/mL
May 2024	Visible haematuria	Flexible cystoscopy: 1.5 cm anterior urethral polyp (at urethral roof, 2-3 cm proximal to meatus) + smaller villous lesion in prostatic urethra
Jul 2024	Rigid cystoscopy & biopsy	Prostatic adenocarcinoma, Gleason 4+4; IHC: PSA+, NKX3.1+, PAX8-, p63- (anterior urethral lesion) & Possible nephrogenic metaplasia (prostatic urethral lesion)
Aug 2024	Staging CT and PSMA PET/CT	Uptake confined to prostate, no nodal/osseous disease
Sep 2024	Endoscopic resection (anterior urethral lesion)	Histology: metastatic prostate adenocarcinoma, PSA/PSAP positive
Ongoing	MDT outcome	Considered isolated urethral recurrence; ADT recommended; patient under PSA and cystoscopy surveillance

## Discussion

Isolated metastasis of prostate cancer to the anterior urethra is an exceedingly rare condition, with only a handful of cases documented in the modern literature [3]. While prostate cancer specifically spreads to bone, lymph nodes, lungs, and liver, a urethral involvement represents a distinctly atypical pattern of dissemination [4,5]. Though penile metastasis in itself is exceptionally uncommon, the urethra is even less frequently affected than the corpus cavernosum or foreskin [4,6]. Reported literature has also highlighted a highly variable interval between primary treatment and recurrence, ranging from only a few months to slightly over a decade [5,6]. 

Several mechanisms have been suggested to elucidate how prostate cancer may spread to the anterior urethra. Direct extension from periurethral prostatic tissue into the lumen is the most widely cited theory, also supported by cases demonstrating continuous tumour growth or extrusion through the meatus [6,7]. Given the frequency of procedures like catheterisation or transurethral resection in this patient population, iatrogenic seeding during such urological instrumentation has been noted as another plausible pathway [1]. Haematogenous and lymphatic dissemination are less typical yet remain credible, especially in aggressive or treatment-emergent variants. Molecular insights further propose that specific genomic alterations, including BRCA2 loss, PTEN/RB1 alterations, and KRAS amplification, may predispose to such unconventional metastatic patterns [4]. Therefore, both biological and mechanical factors complement each other and can be used to explain the pathophysiology of the aforementioned metastasis. 

Clinically, anterior urethral metastases of prostatic cancer most often present with gross haematuria, bleeding per urethra, or visible/meatal nodules [2,5]. Dysuria, along with obstructive voiding symptoms, may also occur, as seen in our case during the initial presentation. A diagnostic challenge that can occur is that PSA levels do not reflect the disease burden. Several cases in the past have described urethral recurrence despite low or undetectable PSA, as also seen in our patient. It has been correlated to poorly differentiated histologies or neuroendocrine features with diminished PSA secretion [2,8]. In the above-described case, disease recurrence was noted notwithstanding previously achieving an undetectable PSA nadir. It is a rare phenomenon that has been previously outlined in several published reports where urethral metastases developed in the absence of concordant PSA elevation [1,3,8]. This definitely underscores the limitations of solitary PSA monitoring and the importance of careful clinical vigilance while detecting such unusual recurrences. Consequently, clinicians need to maintain a level of suspicion if and when novel urethral or lower urinary tract symptoms arise in men with a history of prostate cancer, regardless of biochemical stability. 

Finally, the diagnosis of urethral metastasis relies on a combination of endoscopy, histopathology, and imaging. Urethroscopy or cystoscopy typically reveals a localised lesion, which should then be excised and biopsied for confirmation [1,3]. Furthermore, immunohistochemistry helps in discerning prostatic metastases from primary urethral tumours. Prostatic origin can be strongly instituted by positivity for PSA, prostatic acid phosphatase, and androgen receptor with negativity for CK7/CK20 [1]. Cross-sectional imaging is imperative, not only to confirm the lesion but also to exclude disseminated disease, which significantly alters management [3,5]. 

No standardised treatment has been proven successful or even exists due to the rarity of this presentation. Small and localised lesions have been successfully managed with endoscopic resection; however, larger or distal urethral involvement may require partial urethrectomy or even partial penectomy with reconstruction [2,3]. In unresectable cases or where organ preservation is preferred, radiotherapy can be a viable option [3]. Systemic therapy with androgen deprivation therapy, chemotherapy, or newer hormonal agents tailored to disease biology remains central in patients with castration-sensitive or castration-resistant disease [4]. For more advanced situations, palliative interventions such as local resection or urinary diversion can provide significant symptomatic relief [5]. 

Ranging from durable local control after surgery or radiotherapy to rapid systemic progression despite treatment, outcomes have been noted to be quite heterogeneous in the reported cases. Limited long-term follow-up, complemented by small reported and published case numbers, hinders firm prognostic conclusions. Nevertheless, recurrent observations across the literature that urethral metastases may develop despite low PSA levels, prolonged disease-free intervals, or apparently localised primary tumours emphasise the need for vigilance and individualised care with utmost important multidisciplinary input wherever possible. Our patient’s presentation, with anterior urethral recurrence after several years and also following an undetectable PSA nadir, reinforces these patterns that further add to the limited evidence base. 

## Conclusions

Isolated anterior urethral metastasis from prostate cancer is extremely rare and may present many years after primary treatment. PSA levels can be discordant with disease activity, and recurrence may occur despite an undetectable nadir, making clinical vigilance essential. New urethral symptoms in men with a history of prostate cancer should prompt endoscopic and histological evaluation, even when PSA is reassuring.
